# Supplementary material for: Estimating intraclonal heterogeneity and subpopulation changes from bulk expression profiles in CMap
Source: Life Sci Alliance. 2022 Jun 10;5(10):e202101299. doi: 10.26508/lsa.202101299 (PMC9187873; doi:10.26508/lsa.202101299)
Supplement: Supplementary file 1 [file LSA-2021-01299_Supplemental_Data_1.docx]

Supplementary Materials

Verification by PBMC datasets

To verify the performance of Premnas, we used peripheral blood mononuclear cells (PBMCs) to test the validness of the proposed workflow. First, single cell data of PBMC (GSE127471 ([Newman et al. 2019](#bib_Newman_et_al_2019))), of which dimension were 16476 (genes) X 1054 (cells) after filtering (See **Materials and Methods**), were process by ACTIONet ([Mohammadi et al. 2020](#bib_Mohammadi_et_al_2020)), and totally 9 cell subpopulations and 14 archetypes ([**Fig. S1**](#figS1)) were identified.

With these archetype and subpopulation labels, we further processed PBMC cells by CIBERSORTx ([Newman et al. 2019](#bib_Newman_et_al_2019)). The PBMC bulk dataset we used in digital cytometry, which had also been used to verify CIBERSORTx performance in their own paper, were acquired from CIBERSORTx official website ( [https://cibersortx.stanford.edu/download.php](https://cibersortx.stanford.edu/download.php) ). The hyper-parameters for the PBMC and MCF-7 datasets set in CIBERSORTx were totally same. For example, S-mode correction were both used on both datasets, and the permutation for statistical analysis was also be done (see **Materials and Methods**).

After the digital cytometry, each subpopulation would be annotated by several marker genes (see [**Supplementary Table 1**](#tblS1)) and consequently labeled with corresponding cell type. The annotation was accomplished by ACTIONet function “*annotate.clusters.using.markers*”, which also calculated the confidence score of each annotation. We noticed that the confidence score of subpopulation 9 was significantly lower than others (for subpopulation 1-8, the confidence score were between 15 to 22, while the confidence score of subpopulation 9 was 2.6), and decided to remove subpopulation 9 in the downstream analysis.

Eventually, we compared the composition estimation via digital cytometry with the ground truth composition directly assessed by flow cytometry and the Pearson correlation coefficient between them was high (r = 0.835, see **S1c-d**). This demonstration using PBMC data showed the combination of ACTIONet and CIBERSORTx had reasonable performance across profiling platforms.
